# Coenzyme Q10 supplementation in adult-onset focal segmental glomerulosclerosis caused by the Chinese common pathogenic variant c.737G > A (p.Ser246Asn) in the *COQ8B* gene

**DOI:** 10.1080/0886022X.2025.2501204

**Published:** 2025-05-13

**Authors:** Shi Jin, Yiqin Shi, Pan Lin, Hong Liu, Xialian Xu, Xuantong Dai, Gengru Jiang, Xiaoqiang Ding, Fujun Lin

**Affiliations:** a Department of Nephrology, Zhongshan Hospital, Fudan University, Shanghai Institute of Kidney Disease and Dialysis (SIKD), Shanghai Key Laboratory of Kidney and Blood Purification, Shanghai Medical Center of Kidney Disease, Shanghai, China; b Department of Nephrology, Shanghai Geriatric Medical Center, Shanghai, China; c Department of Internal Medicine, Renal Division, Xin Hua Hospital Affiliated to Shanghai Jiao Tong University School of Medicine, Shanghai, China

**Keywords:** Focal segmental glomerulosclerosis, podocyte, COQ8B, coenzyme Q10

## Abstract

COQ8B nephropathy, a mitochondrial disorder caused by mutations in the *COQ8B* gene, is a major pediatric genetic focal segmental glomerulosclerosis (GFSGS) etiology and stands out as one of the few treatable forms with good response to coenzyme Q10 (CoQ_10_) supplementation. As the diagnosis and clinical experience of COQ8B nephropathy were predominantly in the pediatric population, the long-term efficacy of CoQ_10_ supplementation and its application in the adult-onset patients remains largely unknown. Here, we report three cases of adult-onset FSGS from unrelated families, all carrying the Chinese common *COQ8B* mutation (c.737G > A; p.Ser246Asn) with divergent trajectories of renal function following CoQ_10_ supplementation initiated in different stages of renal dysfunction, providing valuable evidence on the implication of early disease diagnosis and prompt CoQ_10_ supplementation for the prognosis of adult patients affected with COQ8B nephropathy.

## Introduction

Genetic focal segmental glomerulosclerosis (FSGS) refers to a series of rare glomerular diseases resulted from pathogenic mutations in genes related to podocyte cytoskeleton or slit diaphragm. Compared to primary and secondary FSGS, genetic FSGS have higher risk of end-stage renal disease (ESRD) due to early onset, late identification, and lack of targeted therapies. So far, over 60 genes are currently known to be involved in FSGS [1]. Depending on the causative gene, the patients can present with proteinuria, or steroid-resistant nephrotic syndrome (SRNS), and ESRD, or progress to ESRD over the course of 5–10 years. Approximately, 24.0–26.2% of children with SRNS results from a genetic cause [2,3]. In the adult population, genetic causes account for approximately 8–26% of FSGS cases, with collagen type IV-alpha-associated disorders being the most prevalent [4–7].

The *COQ8B* gene (previously referred to as *ADCK4*), located in chromosome 19q13.1, encodes an atypical kinase involved in the coenzyme Q biosynthesis process, which is crucial for podocytes migration. COQ8B nephropathy, an autosomal recessive disease caused by mutation in *COQ8B* gene [8,9], is one of the most common single-gene causative glomerulopathy of pediatric SRNS [10] and is one of the few genetic FSGS form with a targeted therapy available. Oral coenzyme Q10 (CoQ_10_) supplementation was shown to reduce oxidative stress and prevent H_2_S oxidation of podocytes in CoQ_10_ deficiency animal model [11], and was proved effective in proteinuria reduction and kidney function preservation in pediatric SRNS due to primary CoQ_10_ deficiency in a global cohort [12].

COQ8B nephropathy has predominantly been diagnosed and reported in the pediatric FSGS/SRNS cohorts, whereas the long-term efficacy of CoQ_10_ supplementation and its application in adult-onset cases largely unknown. In this study, we present three adult-onset patients from unrelated Chinese families, each exhibiting FSGS on renal pathology and harboring *COQ8B* mutations as confirmed by genetic testing. In addition, we systematically summarized published cases of adult-onset COQ8B nephropathy to provide a comprehensive perspective on the clinical picture and treatment regimens of the disease in the adult population.

## Materials and methods

### Study participants

This study retrospectively collected and analyzed the clinical data, family history, kidney biopsy, and genetic testing from three patients with COQ8B nephropathy who were diagnosed and treated at Zhongshan Hospital, Fudan University and Xinhua Hospital, Shanghai Jiaotong University from June 2019 to October 2024. This study was approved by the Ethics Committee of Zhongshan Hospital, Fudan University (B2021-067R), and Xinhua Hospital, Shanghai Jiaotong University (XHEC-C-2024-185-2). Written informed consent was obtained from the patients for the publication of any potentially identifiable images or data included in this study.

### Renal biopsy

All renal biopsies were evaluated by light microscopy, immunofluorescence, and electron microscopy as routine procedures. All evaluations were examined and reviewed independently by at least two well-trained specialists with rich experience in renal pathology.

### Whole-exome sequencing (WES) and Sanger sequencing validation

Genomic DNA was isolated from peripheral blood lymphocyte and subjected to exome capture using Agilent SureSelect Human All Exon V5 50M kit (Santa Clara, CA) and NimbleGen Technology (Madison, WI) followed by next-generation sequencing on the Illumina HiSeq sequencing platform (San Diego, CA). Candidate variants identified by WES were validated by Sanger sequencing using an ABI PRISM 3730xl Genetic Analyzer (Applied Biosystems, Waltham, MA). Co-segregation of candidate variants was tested in all family members whose DNA sample was available. Variant interpretation was done by a panel of nephrologists or molecular geneticists with domain expertise in inherited kidney diseases, bioinformaticians, and the clinical molecular geneticists, using the American College of Medical Genetics and Genomics (ACMG) guidelines for clinical sequence interpretation [13].

## Results

### Clinical phenotypes

#### Family 1

**Figure 1.**
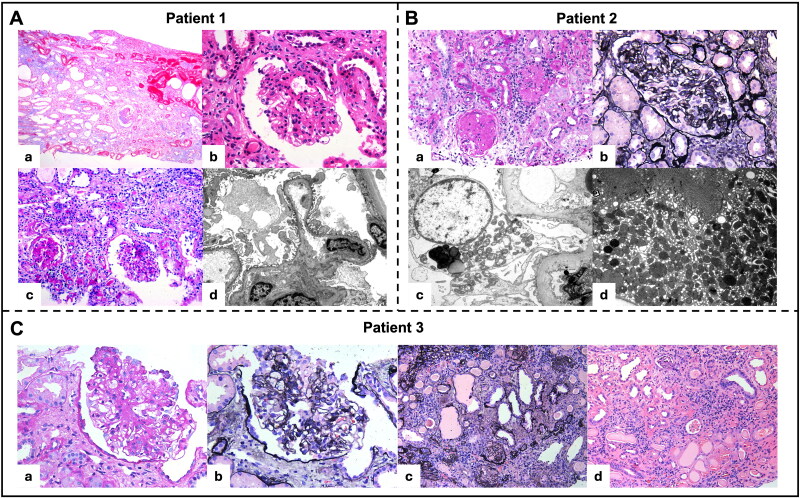
Renal biopsies. Patient 1 (A): renal biopsy showed multifocal tubular atrophy and interstitial fibrosis (a), with one segmental sclerosed glomerulus (b, c). Electronic microscope showed partial foot process effacement (d); patient 2 (B): renal biopsy showed segmental sclerosed glomeruli (a, b). Electronic microscope showed a few dysmorphic mitochondria in podocytes (c) and numerous dysmorphic mitochondria in proximal tubular epithelial cells (d); patient 3 (C): renal biopsy showed a segmentally sclerosed glomerulus (a, b) with the other glomeruli globally sclerosed (c), accompanied by diffuse tubular atrophy and interstitial fibrosis (d).

**Figure 2.**
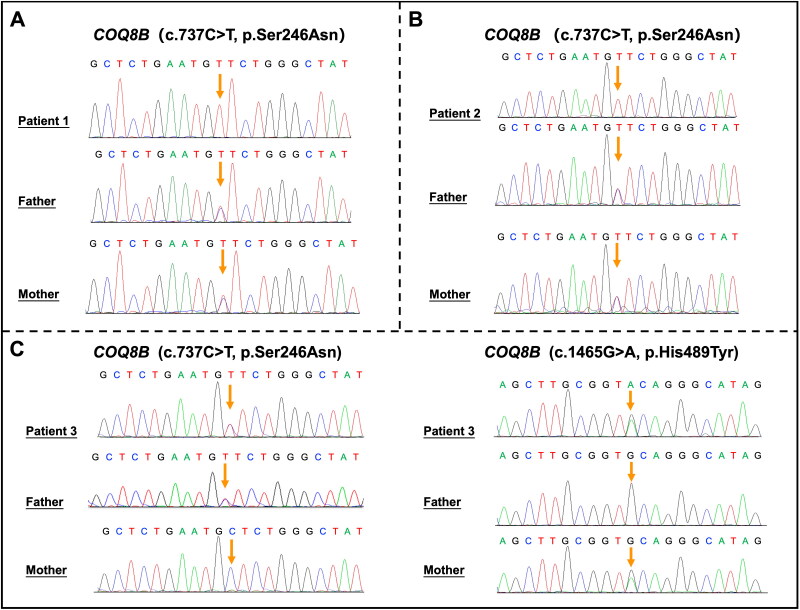
Genotype identification. Patient 1 (A): WES identified a homozygous variant, c.737C > T (p.Ser246Asn) in *COQ8B*. Both parents were asymptomatic heterozygous carriers; patient 2 (B): WES identified a homozygous variant, c.737C > T (p.Ser246Asn) in *COQ8B*. Both parents were asymptomatic heterozygous carriers; patient 3 (C): WES identified compound heterozygous *COQ8B* variants, c.737C > T (p.Ser246Asn) (paternally inherited) and c.1465G > A (p.His489Tyr) (maternally inherited).

Patient 1 was a 23-year-old female who presented asymptomatic proteinuria in routine health checkup. She denied any significant past medical history and family history. Physical examination revealed blood pressure of 117/78 mmHg, pulse rate of 90 bpm with no signs of edema, rash, lymphadenopathy, or organomegaly. Laboratory tests showed a proteinuria level of 3240.6 mg/day, serum albumin of 43.9 g/L, serum creatine of 79 μmol/L, and estimated glomerular filtration rate (eGFR) of 93 mL/min/1.73 m^2^. Glucose, lipid panel, immunology test, tumor screening, and electrophoresis were unremarkable. Ultrasound revealed slightly reduced kidney diameters (88 mm and 94 mm bilaterally). Kidney biopsy showed 44% sclerosed glomeruli, one segmental sclerosed glomerulus, mild mesangial proliferation in the remaining glomeruli, and multifocal tubular atrophy with interstitial fibrosis. Immunofluorescence was negative. Electronic microscope showed 60% partial foot process effacement without electronic dense deposits or glomerular basement membrane changes (Figure 1(A)). WES identified homozygous variant, c.737G > A (p.Ser246Asn) in *COQ8B* with both parents being asymptomatic heterozygous carriers (Figure 2(A)). This variant can be classified as likely pathogenic according to the ACMG criteria (PM2, PP1, PP3, PP4, and PP5). Based on the patient’s clinical and pathological findings, the diagnosis of COQ8B nephropathy was confirmed.

**Figure 3.**
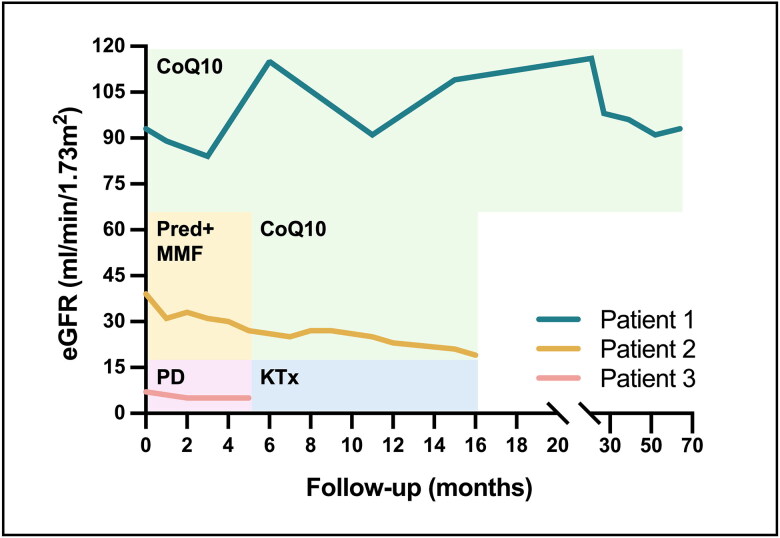
Clinical courses. Changes in estimated glomerular filtration rate was showed for each patient (patient 1, green line; patient 2, yellow line; patient 3, pink line). For each patient, the treatment of CoQ10 (green bar), immunosuppressive agents (yellow bar), peritoneal dialysis (pink bar), and kidney transplantation (blue bar) were depicted. eGFR: estimated glomerular filtration rate; CoQ10: coenzyme Q; Pred: prednisone; MMF: mycophenolate mofetil; PD: peritoneal dialysis; KTx: kidney transplantation.

Patient 1 was immediately started on high-dose CoQ_10_ supplementation. Given that the recommended dose of CoQ_10_ for COQ8B nephropathy in pediatric patients is 15–30 mg/kg/d [12] and due to limited adult experience, CoQ_10_ was initially prescribed at 15 mg/kg/d, with a gradual increase to 30 mg/kg/d. There were no adverse reactions or significant gastrointestinal symptoms leading to discontinuation during a 5-year follow-up. Additionally, valsartan 80 mg/d and dapagliflozin 10 mg/d were prescribed to help minimize proteinuria. Proteinuria rapidly decreased to less than 1.5 g/d after initiating CoQ_10_ treatment, and her eGFR remained stable at 93 mL/min/1.73 m^2^ by the 64th month after diagnosis (Figure 3).

#### Family 2

Patient 2 was a 40-year-old female who presented with elevated blood pressure and foamy urine for a duration of two months. She had a past history of leukoderma but her family history was negative. Physical examination revealed a blood pressure 140/100 mmHg, a pulse rate of 73 bpm with no signs of edema, rash, lymphadenopathy, or organomegaly. Laboratory test showed a proteinuria level of 4580 mg/day, serum albumin of 33 g/L, serum creatine of 145 μmol/L, and eGFR of 39 mL/min/1.73 m^2^. Hemoglobin was 101 g/L. Glucose, lipid panel, immunology test, tumor screening, and electrophoresis were all unremarkable. Ultrasound showed normal kidney size (104 mm and 97 mm bilaterally). Kidney biopsy showed 42% sclerosed glomeruli, two segmental sclerosed glomeruli, mild mesangial proliferation in the remaining glomeruli, and moderate tubular atrophy with interstitial fibrosis. Immunofluorescence was negative. Electronic microscope showed partial foot process effacement with trace mesangial electronic dense deposits (Figure 1(B)).

Initially diagnosed with FSGS, patient 2 received prednisone (Pred) (60 mg/d) combined with mycophenolate mofetil (MMF) (1 g/d). However, her proteinuria and renal function continued to worsen over the following four months, with an eGFR decline of 2.4 mL/min/1.73 m^2^ per month. Genetic testing was subsequently performed, and WES identified a homozygous variant, c.737G > A (p.Ser246Asn) in *COQ8B*. Both parents were asymptomatic heterozygous carriers (Figure 2(B)). Review of electron microscopy revealed a few dysmorphic mitochondria in podocytes, along with numerous dysmorphic mitochondria lacking cristae or with abnormally enlarged cristae in proximal tubular cells. These findings led to the diagnosis of COQ8B nephropathy. Immunosuppressive treatment was gradually tapered and ultimately discontinued. CoQ_10_ supplementation was initiated at 15 mg/kg/d and gradually increased to 30 mg/kg/d. Supportive treatment including dapagliflozin (10 mg/d) and finerenone (10 mg/d) was added during follow-up. This combination of therapies led to partial remission of proteinuria and a reduction in the rate of eGFR decline to 0.73 mL/min/1.73 m^2^ per month by the 16th month of follow-up (Figure 3).

#### Family 3

Patient 3 was a 20-year-old male who presented with fatigue and blurred vision following an influenza infection and recent exposure to nonsteroidal anti-inflammatory drugs two weeks prior. Physical examination revealed a blood pressure of 172/80 mmHg, a pulse rate of 90 bpm with no signs of edema, rash, lymphadenopathy, or organomegaly. Laboratory tests showed a proteinuria level of 8320 mg/day, serum albumin of 32 g/L, serum creatine of 804 μmol/L, and eGFR of 7.4 mL/min/1.73 m^2^. Hemoglobin was 100 g/L. Glucose, lipid panel, immunology test, tumor screening, and electrophoresis were all unremarkable. Ultrasound showed reduced kidney size (85 mm and 84 mm bilaterally).

Kidney biopsy revealed 94% sclerosed glomeruli, with only one segmental sclerosed glomerulus remaining, along with over 90% tubular atrophy and interstitial fibrosis (Figure 1(B)). Immunofluorescence was negative. Electronic microscope confirmed the presence of a sclerosed glomerulus. WES identified compound heterozygous variant, c.737G > A (p.Ser246Asn) and c.1465G > A (p.His489Tyr) in the *COQ8B* gene (Figure 2(C)). The c.1465G > A (p.His489Tyr) variant can be classified as likely pathogenic according to the ACMG criteria (PM2, PM3, PP3, and PP4), supporting the diagnosis of COQ8B nephropathy. Due to end stage renal failure, he underwent peritoneal dialysis (PD) for five months and subsequently received a kidney transplant from his mother (Figure 3). At the one-year follow-up, there was no recurrence of nephrotic syndrome or proteinuria.

## Discussion

Here, we reported three non-syndromic adult patients with negative family history, who presented nephrotic-range proteinuria and FSGS by renal pathology. Genetic testing confirmed the diagnosis of COQ8B nephropathy, caused by the Chinese common pathogenic variant c.737G > A (p.Ser246Asn) in the *COQ8* gene [14–17]. The patients were at varying stages of renal function, leading to diverse treatment strategies and kidney outcomes. This study provides preliminary insight into the application of CoQ_10_ supplementation in adult-onset COQ8B nephropathy and explores its potential long-term efficacy in this population.

*COQ8B* mutations were first reported in adolescence-onset FSGS ad SRNS since 2013 [8]. Advances in molecular genetics and the growing recognition of genetic factors in pediatric nephrotic syndrome have led to an increased detection rate of *COQ8B* mutations, with prevalence estimates ranging from 3.5% to 5.7% in pediatric SRNS [3,10]. Drovandi et al. [12,15] conducted the largest international study of COQ8B nephropathy by collecting data from 140 cases through a systematic literature review and registries queries. Pediatric COQ8B nephropathy is typically characterized by nephrotic range proteinuria, advanced chronic kidney disease (CKD), and extrarenal involvement in approximately 30% of cases. FSGS is the predominant histopathologic finding with uncommon dysmorphic mitochondria in podocytes detectable under electron microscopy. CoQ_10_ supplementation, at doses ranging from 15 mg/kg/d to 30 mg/kg/d, has shown effectiveness in reducing proteinuria and slower eGFR decline in pre-ESRD children.

To date, 32 cases of adult-onset COQ8B nephropathy have been reported [8,9,14,15,18–25]. The clinical data from previous cases are summarized in Table 1 and compared with the pediatric COQ8B nephropathy cohort described by Drovandi et al. [12,15]. Adult-onset COQ8B nephropathy typically manifests at a median age of 24 years (interquartile range: 21–28.5 years). Nephrotic-range proteinuria was present in 30.8% of cases, and 30% of patients were already in ESRD at the time of diagnosis. FSGS was observed in all adult patients who underwent kidney biopsy. CoQ_10_ supplementation was initiated in only nine adult-onset cases, with a relatively small dosage of 9.8 mg/kg/d (interquartile range: 10–25 mg/kg/d) and a shorter duration of follow-up (21 months, interquartile range 12–29 months). Therefore, the long-term efficacy of CoQ_10_ supplementation and its application in the adult-onset patients remains poorly defined. In terms of our study, three adult patients presented with kidney involvement without extrarenal manifestations at the age of 20, 23, and 40. All three exhibited nephrotic-range proteinuria at different stages of CKD: CKD stage 1, 3, and 5, respectively. Notably, the 40-year-old patient represents the latest onset of COQ8B nephropathy ever reported, expanding the age spectrum of this condition. FSGS was observed on all three cases, consistent with previous reports. Oral CoQ_10_ supplementation was initiated in two pre-ESRD patients, leading to a significant reduction in proteinuria and a slower decline in eGFR. By presenting the longest documented observation period for CoQ_10_ supplementation in adult-onset COQ8B nephropathy, our findings suggest that long-term oral CoQ_10_ supplementation at doses of 15 mg/kg/d to 30 mg/kg/d is well tolerated in adult patients, without reported gastrointestinal adverse effects or liver injury. The divergent trajectories of renal function following CoQ_10_ initiation at various stages of renal function provide valuable evidence on the implication of early disease diagnosis and prompt CoQ_10_ supplementation in improving the prognosis of adults affected with COQ8B nephropathy.

**Table 1.** Patient characteristics and clinical outcomes of adult-onset COQ8B nephropathy from published cases, compared to pediatric-onset COQ8B nephropathy from cohort by Drovandi et al.

Patient characteristic	Adult-onset COQ8B nephropathy [8,9,14,15,18–25]	Drovandi et al. [12,15]
Total number of patients (females)	32(20)	140 (65)
Kidney disease presentation		
Age at kidney disease onset, years	24 (21–28.5)	9.9 (5.3–14.4)
Nephrotic-range proteinuria	30.8 (4/13)	71.7 (86/120)
Non-nephrotic range proteinuria	69.2 (9/13)	28.3 (34/120)
CKD stage 1	35 (7/20)	34.3 (35/102)
CKD stage 2–4	35 (7/20)	32.3 (33/102)
ESRD	30 (6/20)	33.3 (34/102)
Renal histopathologic findings		
FSGS	100 (9/9)	77.1 (64/83)
Dysmorphic mitochondria	22.2 (2/9)	10.8 (9/83)
Clinical outcome		
Age at ESRD (years)	22.6 (20.5–24)	13 (10–16.7)
Follow-up (years)	3.4 (2–8)	3.9 (1.3–6.9)
CoQ_10_ supplementation	40.9 (9/22)	42.8 (60/140)
CoQ_10_ dose, mg/kg/d	9.8 (10–25)	20 (15–30)
Observation time on CoQ_10_ supplementation, months	21 (12–29)	24 (12–43.2)
>50% proteinuria reduction	62.5 (5/8)	59 (19/32)
Progression to ESRD during CoQ_10_ supplementation	12.5 (1/8)	12 (7/60)

CKD: chronic kidney disease; ESRD: end-stage renal disease; FSGS: focal segmental glomerulosclerosis; CoQ_10_: coenzyme Q10.

Values are % (number of affected patients/informative number of patients) or median (interquartile range), as appropriate.

Furthermore, patient 3 in our study, who received a renal transplant from his mother carrying the heterozygous *COQ8B* variant, represents the first reported case of living-related donor (LRD) renal transplantation for COQ8B nephropathy. Renal transplantation has been considered feasible for patients with COQ8B nephropathy due to its recessive inheritance pattern. Previous studies have reported a low risk of recurrence following deceased donor renal transplantation in COQ8B nephropathy [16,19,26]. Based on our findings, we suggested that renal transplantation from heterozygous LRD may be a viable option for patients with end stage COQ8B nephropathy.

## Conclusions

We identified three adult-onset COQ8B nephropathy patients with *COQ8B* c.737G > A variant from unrelated Chinese families. These patients exhibited nephrotic range of proteinuria and were at various stages of renal function impairment. Oral CoQ_10_ supplementation, at doses ranging from 15 mg/kg/d to 30 mg/kg/d, proved effective in slowing or preventing kidney failure, particularly when initiated at earlier stages. Renal transplantation from heterozygous LRD was found to be a feasible option for patients with COQ8B nephropathy. These findings underscore the role of mitochondrial dysfunction in glomerular diseases and highlight the importance of personalized therapeutic strategies for genetically defined nephropathy subtypes, with significant implications for both pediatric and adult patient populations.

## Supplementary Material

Outline for original images.pdf

## Data Availability

The datasets used and analyzed during the current study are available from the corresponding author on reasonable request. The data are not publicly available due to privacy or ethical restrictions.
