# Supplementary material for: Coenzyme Q10 supplementation in adult-onset focal segmental glomerulosclerosis caused by the Chinese common pathogenic variant c.737G > A (p.Ser246Asn) in the COQ8B gene
Source: Ren Fail. 2025 May 13;47(1):2501204. doi: 10.1080/0886022X.2025.2501204 (PMC12077437; doi:10.1080/0886022X.2025.2501204)
Supplement: Outline for original images.pdf [file IRNF_A_2501204_SM2008.pdf]

Outline for original images：

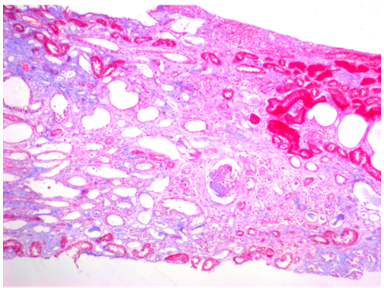

Original Image for Fig1 A a： all included

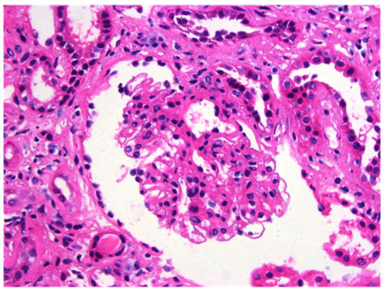

Original Image for Fig1 A b： all included

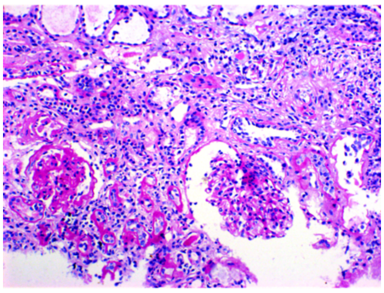

Original Image for Fig1 A c： all included

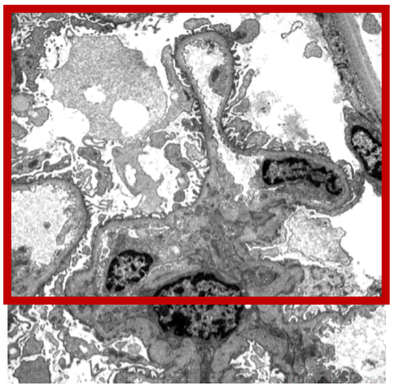

Original Image for Fig1 A d： red box included

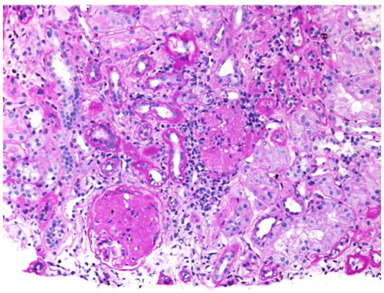
Original Image for Fig1 B a： all included

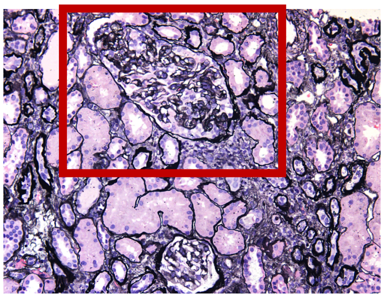
Original Image for Fig1 B b： red box included

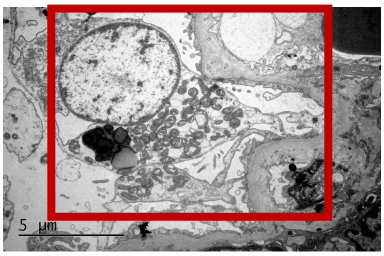

Original Image for Fig1 B c： red box included

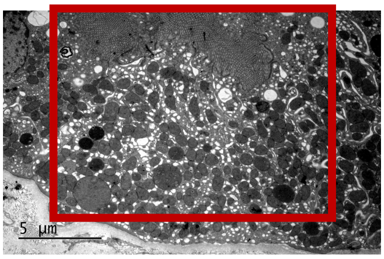

Original Image for Fig1 B d： red box included

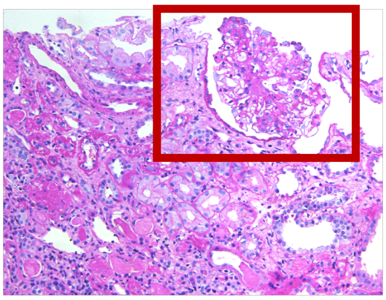
Original Image for Fig1 C a： red box included

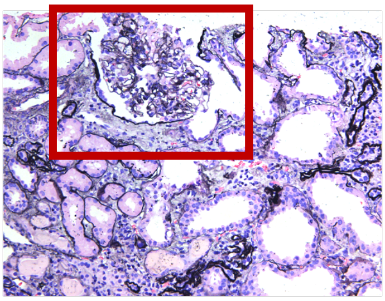

Original Image for Fig1 C b： red box included

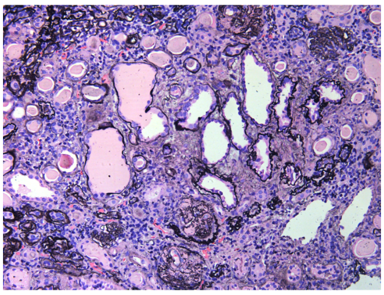

Original Image for Fig1 C c： all included

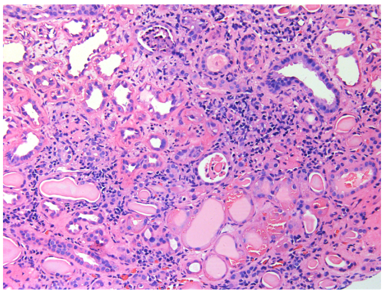

Original Image for Fig1 C d： all included
